# Interpersonal psychotherapy for depression and posttraumatic stress disorder among HIV-positive women in Kisumu, Kenya: study protocol for a randomized controlled trial

**DOI:** 10.1186/s13063-016-1187-6

**Published:** 2016-02-03

**Authors:** Chinwe Onu, Linnet Ongeri, Elizabeth Bukusi, Craig R. Cohen, Thomas C. Neylan, Patrick Oyaro, Grace Rota, Faith Otewa, Kevin L. Delucchi, Susan M. Meffert

**Affiliations:** School of Medicine, University of California, San Francisco, 500 Parnassus Avenue, San Francisco, CA 94121 USA; Kenya Medical Research Institute, PO Box 54840, 00200 Nairobi, Kenya; Department of Psychiatry, University of Nairobi, Kenya, PO Box 19676, Nairobi, Kenya; Department of Obstetrics and Gynecology, University of Nairobi, Kenya, PO Box 19676, Nairobi, Kenya; Department of Obstetrics and Gynecology, University of California, San Francisco, 550 16th Street, San Francisco, CA 94158 USA; Department of Psychiatry, University of California, San Francisco, 4150 Clement Street, San Francisco, CA 94121 USA; Family AIDS Care Education and Services, Kenya, PO Box 614–40100, Kisumu, Kenya; Department of Psychiatry, University of California, San Francisco, 401 Parnassus avenue, San Francisco, CA 94143 USA

**Keywords:** Gender-based violence, Interpersonal psychotherapy, HIV, Major depressive disorder, Posttraumatic stress disorder

## Abstract

**Background:**

Mental disorders are the leading global cause of years lived with disability; the majority of this burden exists in low and middle income countries (LMICs). Over half of mental illness is attributable to depression and anxiety disorders, both of which have known treatments. While the scarcity of mental health care providers is recognized as a major contributor to the magnitude of untreated disorders in LMICs, studies in LMICs find that evidence-based treatments for depression and anxiety disorders, such as brief, structured psychotherapies, are feasible, acceptable and have strong efficacy when delivered by local non-specialist personnel. However, most mental health treatment studies using non-specialist providers in LMICs deploy traditional efficacy designs (T1) without the benefit of integrated mental health treatment models shown to succeed over vertical interventions or methods derived from new implementation science to speed policy change. Here, we describe an effectiveness-implementation hybrid study that evaluates non-specialist delivery of mental health treatment within an HIV clinic for HIV-positive (HIV+) women affected by gender- based violence (GBV) (HIV+ GBV+) in the Nyanza region of Kenya.

**Methods/Design:**

In this effectiveness-implementation hybrid type I design, 200 HIV+ women with major depressive disorder (MDD) and posttraumatic stress disorder (PTSD) who are receiving care at a Family AIDS Care Education and Services (FACES)-supported clinic in Kisumu, Kenya will be randomized to: (1) interpersonal psychotherapy (IPT) + treatment as usual (TAU) or (2) TAU, both delivered within the HIV clinic. IPT will consist of 12 weekly 60-minute individual IPT sessions, delivered by non-specialists trained to provide IPT. Primary effectiveness outcomes will include MDD and PTSD diagnosis on the Mini International Diagnostic Interview (MINI). Primary implementation outcomes will include treatment cost-benefit, acceptability, appropriateness, feasibility and fidelity of the IPT delivery within an HIV clinic.

**Discussion:**

This trial leverages newly defined effectiveness-implementation hybrid designs to gather data on mental health treatment implementation within an HIV care clinic, while testing the effectiveness of an evidence-based treatment for use with a large underserved population (HIV+ GBV+ women) in Kenya.

**Trial registration:**

Clinical Trials Identifier: NCT02320799, registered on 9 September 2014.

## Background

### Global mental health treatment gap in in low and middle income countries (LMICs) and effectiveness-implementation hybrid trials

The mental health care gap in LMICs is well-established – 75 % of those with serious mental disorders never receive care [1]. The majority of those in need of care suffer from depression and anxiety disorders [2], illnesses for which treatments with strong efficacy have existed for decades and are widely used in high income countries (HICs). Over the past decade, efficacy studies have shown that non-specialists can deliver many evidence-based psychotherapies for common mental disorders in LMICs [3], thereby addressing workforce barriers related to the scarcity of mental health professionals. Yet, for the most part, the field has not moved beyond the first (T1) stage of translational research (testing the clinical efficacy of treatments in new settings) to address treatment effectiveness with general populations and implementation strategies. Given the large and growing global magnitude of disability related to mental disorders (a 45 % increase over the past 20 years [4], such research is not only the next logical step for translation, but is required to meet the need for services. In order to move efficiently toward scale-up and implementation of mental health care, we will use an effectiveness-implementation hybrid type I design which has the potential to inform scale-up and implementation of mental health care. As described by Curran and colleagues [5], effectiveness-implementation hybrid designs blend clinical effectiveness and implementation research, and are composed of three categories which shift from an emphasis on effectiveness (type I) to an emphasis on implementation (type III). Type I designs prioritize evaluation of the effectiveness of the intervention, including participant-level randomization, while allowing for data collection on implementation parameters. For example, a type I effectiveness-implementation hybrid design might test the clinical effectiveness of delivering a mental health treatment in a primary care setting and include a multi-stakeholder qualitative process evaluation of the delivery of the intervention.

### HIV, GBV and women: mental health care needs

HIV+ women suffer extraordinarily high rates of gender-based violence (GBV) (GBV + HIV+), particularly child abuse and intimate partner violence [6, 7]. Mental disorders affect most GBV survivors, typically manifesting as anxiety disorders, including posttraumatic stress disorder (PTSD) and depression [8, 9]. Despite the attention received by the HIV-GBV syndemic and awareness that depression and PTSD have a significant negative impact on antiretroviral therapy (ART) adherence [10, 11], few mental health treatment studies assess HIV outcomes. HIV+ GBV+ women in LMICs represent a large subpopulation of those in the mental health treatment gap, who have particularly urgent needs – not only do HIV+ GBV+ women bear a disproportionately heavy mental disorder burden, but the association between mental disorders and ART default creates an urgent mental health treatment need in order to avert HIV-related morbidity and mortality [12].

The preponderance of the world’s HIV-infected populations live in sub-Saharan Africa, where women make up the majority of those infected [13]. Sub-Saharan Africa is also a region with a high lifetime prevalence of GBV, ranging from 56–71 % [14]. Our study site is the Family AIDS Care and Education Services (FACES) HIV Care and Clinical Research clinic in Kisumu, Kenya. FACES is a President’s Emergency Plan for AIDS Relief (PEPFAR)-funded 12-year collaboration between the University of California, San Francisco (UCSF) and the Kenya Medical Research Institute (KEMRI) serving more than 140,000 HIV+ individuals in the Nyanza region of Kenya. This region has the country’s highest national prevalence of HIV (19.3 %) and physical violence against women (57 % of women aged 15–49) [15, 16]. In 2013, we conducted a qualitative mental health care needs assessment of HIV+ GBV+ women served by the FACES linic in Kisumu [17]. While the goal of qualitative studies is not to determine prevalence, but rather to investigate local perceptions within their social context, we found that the majority of interviewees reported that physical violence (79 %, *n* = 27), emotional abuse (82 %, *n* = 28) and sexual violence (76 %, *n* = 26) against HIV+ women were common in the region. Mental health problems among HIV+ GBV+ women included depressive, anxiety, and traumatic stress symptoms and suicidal thoughts. Participants opined that emotional distress from GBV caused HIV treatment default, and led to poor HIV health outcomes even if adherent to an ART regimen. Respondents agreed that mental health treatment was needed for HIV+ GBV+ women and most agreed that the best treatment modality was individual counseling delivered weekly at the HIV clinic. Based on these data, we selected and adapted Interpersonal Psychotherapy (IPT) delivered by local non-mental health specialists within the HIV clinic for treatment of depression and trauma symptoms among female HIV+ GBV+ clinic attendees.

IPT is a time-limited (10–12 weekly sessions) diagnosis-focused psychotherapy, developed in the 1980s by Gerald Klerman and Myrna Weissman to address interpersonal issues in depression [18]. IPT is now a first-line treatment for depression, has the best results in the only face-to-face RCT for depressed HIV+ patients, and is known to be non-inferior to traditional exposure-based psychotherapies for PTSD [19, 20]. IPT intervenes in social functioning with consequent benefits for symptom experience. It helps patients solve an interpersonal crisis characterized as grief, a role dispute, or a role transition, by building social skills and gathering social support [21].

## Design/Methods

### Aims

We will conduct an effectiveness-implementation hybrid type I study of IPT + treatment as usual (TAU) versus TAU alone for major depressive disorder (MDD) and posttraumatic stress disorder (PTSD) among HIV+ GBV+ women receiving HIV care at FACES in Kisumu. IPT will be offered to the TAU group after the conclusion of the 12-week IPT. We will assess the effectiveness of IPT + TAU versus TAU alone for reduction of depression and PTSD symptoms, HIV viral load and improvement of ART adherence and neurocognitive functioning. We will also assess implementation parameters, including cost-benefit analyses of delivering IPT integrated within HIV clinics using non-specialist providers, fidelity and adoption of the intervention. We hypothesize that IPT + TAU will be more effective than TAU alone for remission of MDD and PTSD and that implementation analyses will show that it is delivered with high fidelity and adoption and that the benefits outweigh the costs.

### Setting

The study will be conducted at the FACES HIV Clinic in Kisumu. IPT sessions will take place in a private room in the clinic.

### Study design

This randomized trial will use an implementation hybrid design to assess the effectiveness and implementation parameters of IPT + TAU versus TAU alone for the treatment of PTSD and MDD among HIV+ GBV+ women served by the FACES clinic. IPT will be provided by non-specialists trained to deliver IPT in the HIV clinic setting. The TAU group will be offered IPT treatment following the initial 12-week trial. Thus, all study subjects will receive TAU throughout the study and they will all receive IPT in either the first or second half of the study. Study outcomes will be assessed at baseline, and weeks 12, 24 and 36 by a study evaluator who is blind to group assignment. The study protocol is approved by the UCSF Committee on Human Research and the KEMRI Scientific Ethical Review Unit.

### Participant recruitment, consent, inclusion and exclusion criteria and randomization

#### Recruitment

HIV+ women will be recruited by informing FACES HIV care providers of the study (provider referral) and giving informational talks to FACES patients who are waiting for HIV appointments (self-referral). Consecutive HIV+ women will be consented and screened for inclusion in this study until the desired sample size is reached. Women who do not meet study criteria will be referred to existing FACES psychosocial care and GBV resources as indicated below.

#### Consent

Prospective participants will be invited to meet with study personnel in a confidential room for additional information on the study. If interested, they will then undergo a consent process in which each section will be repeated in their own words to check for understanding (up to three chances). Participants who complete the consent process will then undergo eligibility screening.

#### Inclusion criteria

Participants will be HIV+ women who receive care from FACES-Kisumu, are at least 18 years old, diagnosed with MDD and PTSD secondary to GBV on the Mini International Neuropsychiatric Interview (MINI 5.0), have the ability to attend once a week therapy sessions for 12 weeks and provide written informed consent.

Of note, while the sample size and analyses required to complete a psychometric evaluation of all measures used in our protocol is not within the scope of this study, the MDD and PTSD modules of the widely used, internationally validated MINI [22] were adapted for the local culture and translated into the local languages, Dholuo and Kiswahili, using a standardized process of separate forward and backward translation, with an iterative team approach to resolve discrepancies [23]. This strategy is now widely practiced in the field of global mental health, where separate validation studies for each distinct population have not driven changes in diagnosis or treatment needs, as it was once thought they might [24]. All measures will be read to participants secondary to local literacy rates.

#### Exclusion criteria

Cognitive dysfunction requiring a higher level of care or compromised ability to participate in IPT; severe thought or mood disorder symptoms requiring a higher level of care or interfering with ability to participate in IPT; and current drug and alcohol dependence requiring substance use treatment. Pregnant women are not excluded.

#### Randomization

Blocks of 10–20 participants will be randomized to: (1) IPT + TAU or (2) TAU until reaching the target of 220 participants. The variation in randomization blocks will minimize transparency of assignment and maintain blinding.

### Interventions

IPT is described above.

#### Treatment as usual

At the FACES clinic, TAU resources for HIV+ GBV+ women include medical professionals, community elders, informal counseling, HIV treatment adherence counseling, church leaders, police, and pro-bono legal aid. These providers and services are available for access as needed. No explicit, evidence-based mental health treatment is currently available through TAU, although the social support implicit in each service is known to benefit mental health [25]. At week 12, TAU participants will complete their second assessment and will then be offered the same IPT treatment received by participants in the IPV + TAU arm. Thus, all study subjects will receive IPT during the course of the study.

### Interpersonal Psychotherapy (IPT) therapists

There are no formal education or experience requirements for the community therapists who are trained to deliver IPT in this study. All therapists must be women and all must undergo a 10-day training in IPT, similar to the trainings we have undertaken with other traumatized populations in low-resource settings [26, 27]. Therapists will then complete the run-in phase of the study, during which they will be assigned a pilot IPT case. During the pilot phase and throughout the study, each therapist will receive weekly telephone supervision from one of two psychiatrists who are experts in IPT (study principal investigator (PI) and co-investigator), at which time they will be scored on 10 key aspects of delivering IPT, according to the IPT phase in which they are working and using a 10-point Likert scale for each response. Only those therapists who consistently score “9 s” and “10s” by the end of their IPT case will be allowed to progress to work with participants enrolled in the study trial. Therapists who do not meet these criteria will be provided with one or two additional pilot IPT cases. In addition to weekly individual supervision by psychiatrists who are IPT experts, a train-the-trainer model will be used to provide selected IPT therapists with additional training as on-site peer supervisors, including running weekly, onsite IPT group supervision, to be conducted as an adjunct to ongoing weekly telephone supervision from PI and co-investigator during the trial. Ongoing supervision is recognized as a critical hurdle for successful implementation [28] – the IPT peer group supervision will serve as a key implementation resource for sustaining the intervention following the conclusion of the study.

### Assessments

All study participants in both study arms will be assessed with the same instruments at baseline, 12 weeks, 24 weeks and 36 weeks, with the exception of the trauma history questionnaire, which will only be administered at baseline.

#### Demographics

Demographic data will be collected including age, marital status, ethnic group, ART medications, other mental health treatment and ART counseling received.

#### Effectiveness outcomes

##### Clinical measures

Given the strong association between substance use and trauma-related disorders (“dual-diagnosis”) [29], as well as the impact of the former on overall health, we will measure changes in alcohol and drug use as a secondary outcome for this mental health treatment study of HIV+ GBV+ women. We will use two well-established measures, the Alcohol Use Disorders Identification Test (AUDIT)) [30] and the Drug Abuse Screening Test (DAST) [31]. The AUDIT is a simple 10-question test developed by the World Health Organization to determine if a person’s alcohol consumption over the past 12 months may be harmful. The DAST is a 10-item brief screening tool that assesses drug use, not including alcohol or tobacco use, in the past 12 months.

The Beck Depression Scale (BDI) [32]. The BDI was developed in 1961 and is a 21-item rating inventory that measures characteristic attitudes and symptoms of depression. The BDI is a widely used measure of depression symptoms.

The Conflict Tactics Scale (CTS2) [33]. The CTS was created to measure psychological and physical attacks on a partner in a marital, cohabiting, or dating relationship; and also use of negotiation. The CTS2 is a 39-item revised version of the CTS with expanded evaluation of validity, reliability and specificity.

The Mini International Diagnostic Interview (MINI 5.0) [22]. The MINI is a short, structured diagnostic interview developed in 1990 by psychiatrists and clinicians in the United States and Europe for *Diagnostic and Statistical Manual of Mental Disorders, fourth edition* (DSM-IV) and *International Classification of diseases, 10th edition* (ICD-10) psychiatric disorders. The MINI has multiple modules. This study uses the depression and PTSD modules. The MINI can be administered by lay personnel and takes an average of 15 minutes to complete.

The Posttraumatic Stress Disorder Checklist-Civilian (PCL-C) [34]. For this study, we used the PCL which is a 17-item measure of DSM-IV symptoms of PTSD.

The Social Adjustment Scale (SAS) [35]. The SAS is a 54-item scale that assesses behavioral and emotional social adjustment across six major areas: work, leisure, extended family, primary relationship, parental and family unit.

The Trauma History Screen (THS) [36]. The THS questionnaire is a 24-item self-report measure that examines experiences with potentially traumatic events such as crime, major life events, and sexual and physical assault using a yes/no format.

The World Health Organization Quality of Life Brief (WHOQOL-BREF) [37]. The WHOQOL-BREF was developed in 1991. It comprises 26 items, which measure the following broad domains: physical health, psychological health, social relationships, and environment.

The World Health Organization Disability Assessment Schedule (WHODAS) 2.0 [38]. The WHODAS 2.0 is a 36-item instrument based on the International Classification of Functioning, Disability and Health (ICF), that measures general health and disability levels, including mental and neurological disorders.

##### Neurocognitive testing

With our collaborators in neurology who have separately adapted and validated neurocognitive testing for patients at the FACES clinic [39, 40], we will assess neurocognitive domains affected by HIV-associated impairment: executive function; psychomotor function; attention/speed of information processing; visual and verbal learning; visual and verbal memory. The neurocognitive battery was adapted, translated and normalized for use with FACES HIV clinic patients and paraprofessionals were trained to administer the tests. The battery includes: Category Fluency (animals and first names) [41], Color Trails 1 and 2 [42], Finger Tapper [43], Grooved Pegboard [44], WAIS-III Digit Symbol [45], Digit Span Subtests [42], Brief Visual Memory Test (BVMT) [46], World Health Organization/University of California, Los Angeles (WHO/UCLA), Auditory Verbal Learning Test (AVLT) [47], BVMT Delayed Recall [46], and WHO/UCLA AVLT Delayed Recall [48]. Study personnel were trained to conduct neurocognitive testing by the neurologist and PI of the collaborating team, using the same methodology which she uses in her separate neurocognitive research at the same clinic, with ongoing fidelity and supervision.

##### HIV treatment adherence

The Visual Analog Scale (VAS) will be used for self-report of adherence to ART [49]. Viral load testing will be used as a biological measure of adherence. Blood draws will be performed by a trained phlebotomist at the FACES HIV clinic. Samples will be tested using the Roche TaqMan HIV viral load platform at the KEMRI Center for Disease Control (CDC) HIV-R laboratory. The lowest limit of detection for the TaqMan platform is 20 copies/ml. Therefore, if the VL is below the detection limit, the result will be less than 20 copies/ml and coded as “undetectable.” All other results will have numerical values.

#### Implementation outcomes

##### Acceptability and appropriateness

The acceptability and appropriateness of IPT for treatment of MDD and PTSD in HIV+ GBV+ women will be assessed through semi-structured closing interviews and focus groups with participants, IPT therapists, clinic personnel and members of the FACES Community Advisory Group (CAG).

##### Domains of interest

*Participants* will be asked: (1) to identify positive and negative aspects of IPT, including content, delivery, logistics; (2) if they would refer other people to the treatment and why; (3) to identify suggestions for improvements. *IPT therapists* will be asked: (1) if they believed IPT benefitted participants and why; (2) whether they would like to continue providing IPT; and (3) what suggestions they have to improve the intervention. *Clinic personnel and members of the clinic*’*s CAG* will be asked: (1) to identify positive and negative aspects of IPT, including delivery, logistics; (2) to identify positive and negative impacts of treatment on the HIV clinic and community; (3) whether or not they would like continuation of IPT treatment for MDD and PTSD for HIV+ GBV+ women in their clinic/community and why/why not; (4) suggestions for improvement of either treatment. Four focus groups will be conducted with five participants each (*n* = 20). Focus groups will include: (1) study participants in IPT; (2) IPT therapists; (3) primary care personnel; and (4) CAG members. Ten individual interviews (*n* = 10) will be conducted with participants, providers, primary care clinic personnel and CAG members.

##### Feasibility

The treatment will be considered feasible if the 220 study participants are recruited by screening at least 300 HIV+ GBV+ women, if each completer attends ≥80 % of IPT sessions, attrition is 50 or fewer study participants and if 50 % or fewer of the study therapists voluntarily drop out of the training course and trial.

##### Fidelity

As mentioned above, therapists will discuss each IPT session with an IPT supervisor by telephone and peer IPT supervision group onsite. During supervision, therapists will be assisted with their adherence to and application of IPT. Adherence will be scored using a 10- point Likert scale assessing adherence in each of the three IPT phases. Sessions will be considered adherent to the IPT protocol if they average a score of 5 or higher and do not employ off-protocol interventions. All sessions will be audio-taped and 20 % of sessions will be selected using blocked randomization to ensure both early and late sessions are included, then transcribed and translated for IPT adherence scoring by an independent rater.

##### Economics

The Sustainable East Africa Research on Community Health (SEARCH) economic assessment is a standardized questionnaire developed by Dr. Harsha Thirumurthy for use with HIV-affected populations in sub-Saharan Africa. The instrument assesses both formal (e.g., salaries, wages) and informal (e.g., crops, animals) income in order to accurately capture economic activity in the target population.

### Sample size

As an effectiveness-implementation hybrid type I, the primary goal of this study is to assess effectiveness, while also collecting data on implementation parameters [5]. In our prior work using a similar treatment with a traumatized, low-resource population, we observed a Cohen’s *d* effect size of 0.79 for depression symptoms [20]. Using the depression Cohen’s *d* would suggest that a sample size of at least 50 is necessary for a two-group design with power of 50 and probability level of 0.05. However, given cultural differences between studies, and that this study requires participants to complete four assessments across 36 weeks, with substantial time investment at each assessment, we have made a conservative sample size estimate, allowing for approximately 20 % general attrition at each assessment point, a sample size of 100 was selected. Given the multiple blood draws required of participants and the evidence suggesting that fear of blood draw can lead to attrition [50], we will further increase our sample size to 200 patients.

### Data management

Data collection will be done with paper and tablets. Informed consent will be in paper format, allowing participants to either sign or use a thumbprint to acknowledge receipt of information. Eligibility screening and measures will be collected on Android tablets using an electronic data capture, CommCare Open Data Kit (ODK). ODK is widely used in low- resources setting, is user-friendly and allows users to input data offline, then upload through a secure server. In addition, all screening and therapy sessions will be audiotaped and uploading into REDCap. REDCap is a secure, web-based application designed to support data capture for research studies, providing: (1) an intuitive interface for validated data entry; (2) audit trails for tracking data manipulation and export procedures; (3) automated export procedures for seamless data downloads to common statistical packages; and (4) procedures for importing data from external sources. Audio sessions will be randomly selected for review by an independent rater for external validity.

### Statistical analysis

#### Effectiveness

The main analysis will consist of comparing the change from baseline to post-treatment (12 weeks) between the two treatment conditions. The two primary outcome measures of PTSD and MDD diagnosis will be analyzed by estimating and testing a statistical model of each dichotomous measure with a binomial distribution and log link using generalized estimating equations to account for clustering of participants by therapist (therapist effect) and the repeated assessments. Maintenance of gains will be assessed by testing for significant change from 12-week to 24- and 36-week follow-up assessments within each condition using McNemar’s test and repeated measures *t* tests.

#### Implementation

##### Feasibility

The treatment will be considered feasible if the 200 study participants are recruited by screening at least 300 HIV+ GBV+ women, if each completer attends ≥80 % of IPT sessions, attrition is fewer than 50 study participants and if one or fewer therapists voluntarily drop out of the training course and trial.

##### Acceptability

Interview material will be recorded in audio files, transcribed and translated. Dedoose software will be used to analyze the resulting text using grounded theory. Grounded theory sorts data in three ways: through concepts, categories and propositions. Concepts are identified by seeking conceptual similarities among focus groups/interviews. These concepts are grouped to develop categories of concepts. Categories are then compared and grouped to develop propositions or theoretical constructs about the phenomena of interest. Dedoose software facilitates coding of observations to work toward the theoretical construct. Data will be collected using a “triangulation” technique that compares information from various sources (focus groups, individual interviews) in order to increase the reliability and validity of the findings.

##### Feasibility and fidelity

Feasibility and Fidelity measures will be assessed by sample means and proportions, with 95 % confidence intervals, and compared to pre-specified benchmarks (see above).

##### Cost-benefit analyses

We will use data obtained using the SEARCH economic assessment on the income status of those treated to calculate the economic benefits associated with IPT + TAU versus TAU. The value of these economic benefits will be summarized on a per-person basis and then combined with the cost data to generate a rate or return on IPT + TAU treatment of HIV+ GBV+ women at the FACES clinic.

## Discussion

While the treatment gap for mental health care has been recognized for nearly 20 years, with a few exceptions [51] the majority of studies addressing this gap have used efficacy designs without the benefit of implementation science. This trial will leverage newly defined effectiveness-implementation hybrid designs [5] to gather data on treatment implementation within an HIV care clinic, while testing the effectiveness of an evidence-based treatment for use with a large underserved population (HIV+ GBV+ women) in Kenya. This trial is likely to yield findings that will be generalizable beyond the study population and, if effective, could be scaled up for treatment of HIV+ women in Kenyan HIV clinics and perhaps other countries in sub-Saharan Africa facing a similar HIV-GBV syndemic among women.

Despite awareness of the relationships between GBV, mental disorders and HIV outcomes, this is the first study of which we are aware that intervenes on mental disorders among HIV+ GBV+ women and also collects data on HIV and neurocognitive outcomes. We expect these secondary outcomes to inform women’s HIV care in the region and to shed light on the mechanisms of ART deficits and re-victimization commonly observed among HIV+ GBV+ women.

We recognize a number of limitations in this study. First, generalizability may be limited given that this is a single center study; future multi-center studies will be needed. Another limitation of this study is the TAU control. While TAU includes access to mental health care services, data bearing on the particular efficacy of IPT would be stronger if an active control with similar meeting schedule was included (e.g., weekly, individual supportive therapy). However, given that this study is not intended to test the efficacy of IPT, which has already been established across diverse populations and disorders, but rather to evaluate its effectiveness with a more general and diverse population (HIV+ GBV+ women with MDD and/or PTSD) when implemented by non-specialists within an HIV clinic, we have elected to focus resources on data that will inform scale-up of care.

### Trial status

The trial is actively recruiting participants.

## Abbreviations

ART: antiretroviral therapy
AUDIT: Alcohol Use Disorders Identification Test
AVLT: Auditory Verbal Learning Test
BDI: Beck Depression Scale
BVMT: Brief Visual Memory Test
CAG: Community Advisory Group
CDC: Center for Disease Control
CTS2: Conflict Tactics Scale
DAST: Drug Abuse Screening Test
FACES: Family AIDS Care Education and Services
GBV: gender-based violence
HICs: high income countries
HIV: human immunodeficiency virus
IPT: Interpersonal Psychotherapy
KEMRI: Kenya Medical Research Institute
LMICs: low and middle income countries
MDD: major Depressive Disorder
MINI: Mini International Neuropsychiatric Interview
ODK: CommCare Open Data Kit
PCL-C: Posttraumatic Stress Disorder Checklist-Civilian
PTSD: posttraumatic stress disorder
SAS: Social Adjustment Scale
SEARCH: Sustainable East Africa Research on Community Health
TAU: treatment as usual
THS: Trauma History Screen
UCLA: University of California, Los Angeles
UCSF: University of California, San Francisco
VAS: Visual Analog Scale
WHO: World Health Organization
WHODAS: World Health Organization Disability Assessment Schedule
WHOQOL-BREF: World Health Organization Quality of Life Brief
