# Erratum to: ‘Interpersonal psychotherapy for depression and posttraumatic stress disorder among HIV-positive women in Kisumu, Kenya: study protocol for a randomized controlled trial’

**DOI:** 10.1186/s13063-016-1289-1

**Published:** 2016-03-21

**Authors:** Chinwe Onu, Linnet Ongeri, Elizabeth Bukusi, Craig R. Cohen, Thomas C. Neylan, Patrick Oyaro, Grace Rota, Faith Otewa, Kevin L. Delucchi, Susan M. Meffert

**Affiliations:** School of Medicine, University of California, San Francisco, USA; Kenya Medical Research Institute, Nairobi city, Kenya; Department of Obstetrics and Gynecology, University of Nairobi, Nairobi, Kenya; Department of Obstetrics and Gynecology, University of California, San Francisco, USA; Department of Psychiatry, University of California, San Francisco, USA; Family AIDS Care Education and Services, Kisumu, Kenya

Unfortunately, the original version of this article [1] contained an error. There was an error in one of the author’s affiliations for this publication. Dr Elizabeth Bukusi should only be listed as affiliated with the Department of Obstetrics and Gynecology, University of Nairobi, Kenya.
